# Phenotypically heterogeneous podoplanin-expressing cell populations are associated with the lymphatic vessel growth and fibrogenic responses in the acutely and chronically infarcted myocardium

**DOI:** 10.1371/journal.pone.0173927

**Published:** 2017-03-23

**Authors:** Maria Cimini, Antonio Cannatá, Gianandrea Pasquinelli, Marcello Rota, Polina Goichberg

**Affiliations:** 1 Department of Anesthesiology, Perioperative and Pain Medicine, Brigham and Women’s Hospital, Harvard Medical School, Boston, Massachusetts, United States of America; 2 Unit of Surgical Pathology, Department of Experimental, Diagnostic and Specialty Medicine (DIMES), S. Orsola-Malpighi Hospital, University of Bologna, Bologna, Italy; University of Central Florida, UNITED STATES

## Abstract

Cardiac lymphatic vasculature undergoes substantial expansion in response to myocardial infarction (MI). However, there is limited information on the cellular mechanisms mediating post-MI lymphangiogenesis and accompanying fibrosis in the infarcted adult heart. Using a mouse model of permanent coronary artery ligation, we examined spatiotemporal changes in the expression of lymphendothelial and mesenchymal markers in the acutely and chronically infarcted myocardium. We found that at the time of wound granulation, a three-fold increase in the frequency of podoplanin-labeled cells occurred in the infarcted hearts compared to non-operated and sham-operated counterparts. Podoplanin immunoreactivity detected LYVE-1-positive lymphatic vessels, as well as masses of LYVE-1-negative cells dispersed between myocytes, predominantly in the vicinity of the infarcted region. Podoplanin-carrying populations displayed a mesenchymal progenitor marker PDGFRα, and intermittently expressed Prox-1, a master regulator of the lymphatic endothelial fate. At the stages of scar formation and maturation, concomitantly with the enlargement of lymphatic network in the injured myocardium, the podoplanin-rich LYVE-1-negative multicellular assemblies were apparent in the fibrotic area, aligned with extracellular matrix deposits, or located in immediate proximity to activated blood vessels with high VEGFR-2 content. Of note, these podoplanin-containing cells acquired the expression of PDGFRβ or a hematoendothelial epitope CD34. Although Prox-1 labeling was abundant in the area affected by MI, the podoplanin-presenting cells were not consistently Prox-1-positive. The concordance of podoplanin with VEGFR-3 similarly varied. Thus, our data reveal previously unknown phenotypic and structural heterogeneity within the podoplanin-positive cell compartment in the infarcted heart, and suggest an alternate ability of podoplanin-presenting cardiac cells to generate lymphatic endothelium and pro-fibrotic cells, contributing to scar development.

## Introduction

The cardiac lymphatic system is crucial for the control of intra-myocardial pressure and prevention of swelling, lipid transport, and balanced regulation of tissue inflammation (reviewed in [1–4]). Although little is known about the distribution and activity of cardiac lymphatic vessels (CLVs), there is a documented link between lymphatic malfunction and cardiovascular diseases, including post-MI edema, fibrosis and scarring, and the evolution of congestive heart failure [3, 5–9].

Based on the hitherto reported data, the adult cardiac lymphatic vasculature consists of a network of sub-epicardial and sub-endocardial vessels and a plexus of myocardial capillaries of various diameters and variable concentrations in the different regions of the heart [2–4, 10, 11]. By employing immunohistochemical labeling of proteins preferentially expressed in lymphatic endothelial cells, such as lymphatic vessel endothelial hyaluronan receptor-1 (LYVE-1), membrane glycoprotein podoplanin, prospero homeobox-1 (Prox-1) transcription factor, or vascular endothelial growth factor-3 (VEGFR-3), it was established that the localization and morphology of CLVs are substantially altered in pathological conditions [7–9, 11–15]. Acutely after MI, the density of CLVs increases in the early phases of wound granulation and is further elevated at later stages of tissue repair, superseding the number of blood vessels (BVs) in the scar [9, 12–15]. The post-MI lymphangiogenesis in the human heart is mostly apparent in the scar, infarct border zone (BZ) and reactive pericarditis [9]. Likewise, in murine models of MI, the development of new CLVs is primarily detected in severely damaged myocardium and the adjacent BZ [13–16]. There is evidence that CLVs are involved in adverse ventricular remodeling [13], potentially promoting the maturation of fibrosis and formation of a stable scar [12, 16]. Yet, experimentally-induced impairment in cardiac lymph flow leads to exacerbated and prolonged inflammation after MI [1, 5], and promoting post-MI lymphangiogenesis is suggested to facilitate structural and functional recovery of the mouse [14] and rat [15, 17] hearts. Thus, lymphangiogenic processes in the infarcted heart may have pleiotropic effects on the fibrogenic responses and scar maintenance.

Importantly, the cellular sources of the CLVs in the healing MI remain to be revealed. In order to recognize putative cell populations participating in post-MI lymphangiogenesis and fibrosis during different phases of wound repair, we performed characterization of the distribution of an established lymphendothelial epitopes, podoplanin, LYVE-1, Prox-1 and VEGFR-3, along with the analysis of cell phenotypic markers associated with angiogenic and fibrogenic responses, including CD34, platelet-derived growth factor receptor (PDGFR) α and PDGFRβ, vimentin, and α-smooth muscle actin (α-SMA). Our data point to an unexpected heterogeneity in the podoplanin-positive cardiac cell compartment, which might be significant for the processes of CLV growth after injury, development of fibrosis and scar maintenance.

## Materials and methods

### Myocardial infarction

Experiments were conducted according to the NIH Guide for the Care and Use of Laboratory Animals and were approved by the Brigham and Women’s Hospital Institutional Animal Care and Use Committee (IACUC). C57BL/6 mice (Charles River Laboratories) and BDF1 Kit/GFP transgenic mice [18] (bred in house) behaved similarly and were used interchangeably with identical results. We have elected to employ female mice to reduce biological variability related to the sex of animals. In this regard, previous studies have documented that female mice exhibit better survival and succumb less to heart failure after myocardial infarction (MI) compared to male mice [19, 20]. Future investigations will establish whether lymphatic vessel growth and fibrogenic responses in the ischemic heart are comparable in male and female animals. MI was induced at 2–3 months of age as follows: animals were anesthetized with isoflurane 1.5% and ventilated; under sterile conditions the thorax was opened via the third costal space, the atrial appendage elevated, the left coronary artery located, and a silk braided suture (6–0) was inserted and tightened around the vessel near the origin. Then, the chest was closed and pneumothorax reduced by negative pressure, and the animals were allowed to recover. Sham-operated (SHAM) mice were subjected to an identical surgery procedure, with the exception that the suture was not tightened around the artery. Non-operated (NO) mice served as additional controls. At the time of sacrifice, with the animals under deep anesthesia, bilateral thoracotomy was performed, the hearts were removed and either fixed and processed for histological analysis, or enzymatically digested [21] for single-cell assessment by flow-cytometry, as described below.

### Immunohistochemistry of thin cardiac sections

Hearts were perfused with 10% formalin, fixed and embedded in paraffin. Cardiac tissues were cut into 4 μm-thick sections. Following deparaffinization, rehydration, and heat-induced antigen retrieval (pH 6.0), samples were indirectly immunolabeled with commercially-available primary antibodies and corresponding fluorophore-conjugated secondary reagents; a complete list of antibodies is provided in the S1 Table. Nuclei were counterstained with Hoechst 33342 (Life Technologies) or 4',6-diamidino-2-phenylindole dihydrochloride (DAPI; Sigma-Aldrich). Multiple sections from the hearts of at minimum 4 mice for each time point after MI and 3 mice per sham-operated group were examined, and representative micrographs are included in the figures. Images were acquired with Olympus FluoView FV100 laser scanning confocal microscope equipped with CCD camera (Bio-Rad), using 20X, 40X and 60X objectives. Optical sections (ΔZ = 0.5 to 1 μm) spanning the sample thickness were projected into a single plane for each color channel and merged using Adobe Photoshop (Adobe) or Imaris (Bitplane) software. Alternatively, the sections were blocked with hydrogen peroxide and indirectly immunolabeled with MOMA-2 or F4/80 antibodies (see S1 Table), followed by the development with diaminobenzidine (DAB) substrate kit (Vector) and counterstaining with hematoxylin and eosin (Poly Scientific R&D Corp.). Images were acquired using Olympus BX63 light microscope (Olympus Scientific Solutions Americas) with 20X and 40X objectives and assembled in Adobe Photoshop. Quantitative image analysis was performed with NIH ImageJ by scoring multiple imaging fields of 0.4 mm^2^ (20X objective) and 0.045 mm^2^ (60X objective) for every indicated time point after MI in the scar and border zone (BZ) and remote area (RA) as follows: Podoplanin labeling was measured as % area above binary threshold of positive pixels out of total area populated by cells. Podoplanin co-labeling with LYVE-1, CD34 and VEGFR-3 was calculated using JACoP (Just Another Colocalization Plugin) to determine the degree of co-localization (ranging from the minimum of “0” to maximum of “1”) by Manders overlap coefficient, i.e., the fraction of intensity in a channel of interest located in the pixels displaying above a threshold signal in the podoplanin channel. The occurrence of Prox-1, PDGFRα or PDGFRβ staining in podoplanin-positive cells was assessed by counting the % of double-labeled cells from the total number of podoplanin-positive cells in the imaging field.

### Immunolabeling of thick cardiac sections

Hearts were perfused with 4% paraformaldehyde and stored at 4°C. Sections of 75 to 250 μm were prepared using Leica VT1200 vibrating blade microtome (Leica Biosystems), and indirectly immunolabeled employing the reagents detailed in the S1 Table, and Alexa Fluor 647-conjugated isolectin GS-IB4 (Life Technologies). Images were acquired with Olympus FluoView FV100 laser scanning confocal microscope using 10X and 20X objectives. Optical sections (ΔZ = 1.5 to 2.5 μm) were projected into a single plane for each color channel, and merged using Adobe Photoshop or Imaris software. Representative micrographs are included in the figures.

### Flow-cytometry analysis of isolated cardiac cells

Infarcted (MI) and sham-operated (SHAM) C57BL/6 mice were euthanized at 2 days after surgery, as described above. Non-operated (NO) age-matched animals were used as controls. The hearts were excised and extensively washed in phosphate buffered saline. The cardiac tissues were minced and subjected to repetitive rounds of enzymatic digestion with collagenase type 2 (Worthington Biochemical Corp.) until complete dissociation. Larger cells, such as mature myocytes, were precipitated, and the supernatants containing small cell populations were filtered through 40 μm cell strainers. High cell viability after isolation (~98%) was confirmed by flow-cytometry based on 7-AAD (BD Biosciences) exclusion. Samples were then either immediately stained with podoplanin and VEGFR-3, or fixed in 4% paraformaldehyde and immunolabeled for podoplanin only, or podoplanin in conjunction with either LYVE-1, PECAM-1, CD34, Ly6C, CD11b, F4/80, PDGFRα or PDGFRβ. Prox-1 labeling was performed after the incubation of unfixed cells with podoplanin antibody, using fixation and permeabilization reagents from the transcription factor staining buffer set (affymetrix eBioscience) according to manufacturer’s instructions. The antibodies used for flow-cytometry are listed in the S1 Table. Non-immune normal goat, rabbit, syrian hamster and rat IgGs and isotype controls (detailed in the S1 Table) were employed as negative controls for the respective antigen-specific labeling. Similar procedures for mouse cardiac cell isolation and antibodies for the detection of podoplanin, LYVE-1, F4/80 or PDGFRα by flow-cytometry, were recently reported by other groups [22, 23]. Samples were acquired with BD FACSCantoII (BD Biosciences) and analyzed using FlowJo software (Tree Star Inc.). Single cells were gated using FSC-A/SSC-A followed by FSC-H/FSC-W and SSC-H/SSC-W in all experiments. Compensation settings, gating of positive populations and calculations of % positive cells were performed based on non-immune and isotype IgGs and fluorescence minus one controls.

### Statistical analysis

Data were presented as values for individual mice and means. Statistical analysis was performed with two-tailed *t*-test or one-way ANOVA and Tukey’s *post hoc* test for multiple comparisons using GraphPad Prism (GraphPad Software).

## Results

### Time-dependent increase in podoplanin expression in the infarcted heart

**Fig 1:**
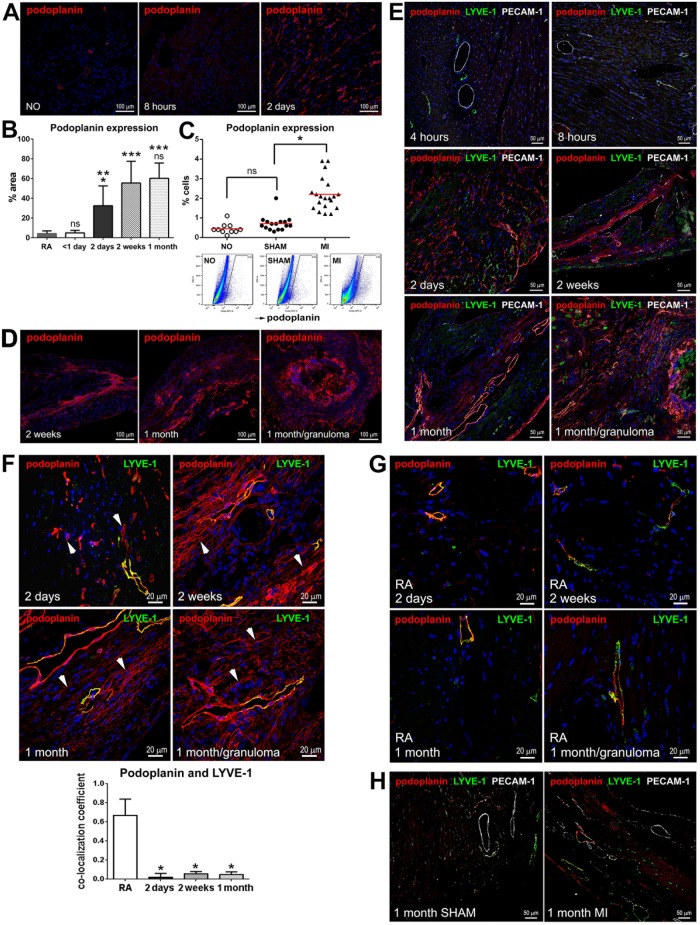
Podoplanin expression in the infarcted and non-infarcted hearts. (**A**) Thin cardiac sections from non-operated (NO) mice and animals at the indicated times after MI were indirectly immunolabeled with podoplanin (red). Nuclei, blue. Areas neighboring the necrotic myocardium are shown for the infarcted hearts. Note the increase in podoplanin immunoreactivity at 2 days after MI. (**B**) Quantitative image analysis of the changes in podoplanin immunolabeling in thin cardiac sections in the infarcted myocardium at the indicated times after MI. RA, remote area. Data represent mean and SD of % area stained with podoplanin; n = 6–10 image fields per group. By one-way ANOVA, *P < 0.02 for 2 days vs. RA, 2 weeks, or 1 month; **P = 0.0017 for 2 days vs. < 1 day; ***P < 0.0001 for RA vs. 2 weeks or 1 month and for < 1 day vs. 2 weeks or 1 month; ns, non-significant for < 1 day vs RA, and for 1 month vs. 2 weeks. (**C**) Flow-cytometry analysis of the frequency of podoplanin-positive cells in the hearts of non-operated (NO), and the sham-operated (SHAM) and infarcted (MI) mice at 2 days after surgery. Graph depicting data from individual animals (upper row) and representative flow-cytometry scatterplots (lower row) are shown. n = 10–20 animals per treatment; mean values are represented by the red line on the graph. By one-way ANOVA, *P < 0.0001 for MI vs. SHAM or NO; ns, not significant for SHAM vs. NO. (**D**-**H**) Thin cardiac sections obtained at the indicated times after MI were indirectly immunolabeled with antibodies that recognize podoplanin (**D**-**H**; red), LYVE-1 (**E**-**H**; green), and PECAM-1 (**E** and **H**; grey). Nuclei, blue. Corresponding single channel images (**E**-**H**) are included in S1 Fig. Areas affected by ischemia are depicted in **D**-**F**; Remote area (RA) is shown in **G**. SHAM, sham-operated in **H**. In **F**, the arrowheads in representative images (upper panels) point to the examples of podoplanin-positive LYVE-1 negative cells. Note the accumulation of LYVE-1-positive CLVs (red and green) as well as podoplanin-expressing cells lacking the LYVE-1 labeling (red only) in the infarcted myocardium as opposed to RA and SHAM. Quantitative image analysis (lower panel) of the podoplanin co-labeling with LYVE-1 is included in the graph. Data represent mean and SD of the co-localization coefficient measured at indicated times after MI and the remote area (RA); n = 5–10 image fields per group. By one-way ANOVA, *P < 0.0001 for RA vs. 2 days, 2 weeks or 1 month; no significant changes between 2 days and 2 weeks and 1 month.

**Fig 2:**
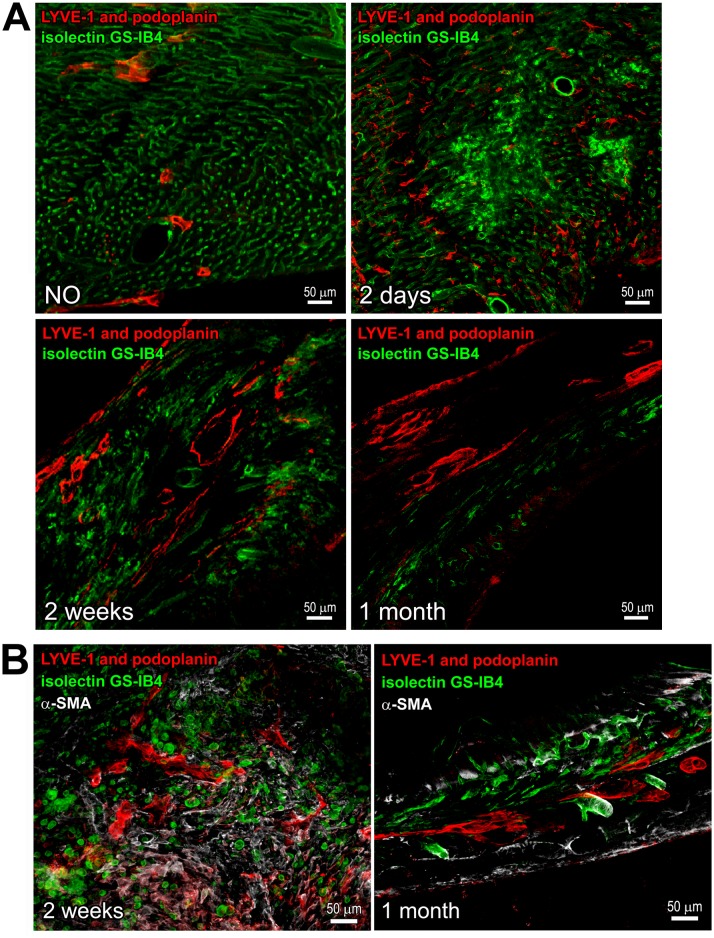
CLVs in the forming and mature scar. Thick cardiac sections were indirectly immunolabeled with the mix of LYVE-1 and podoplanin antibodies (**A**,**B**; red) and α-SMA antibody (**B**; grey), and co-stained with isolectin GS-IB4 (**A**,**B**; green). NO, non-operated. Time after MI is indicated. Corresponding single channel images are included in S2 Fig. CLVs are recognized by the staining with podoplanin and LYVE-1. In **A**, note the changes in the abundance and distribution of the vessels and LYVE-1 and podoplanin immunolabeled cells at different stages of infarct healing. In **B**, α-SMA-positive cells are apparent in the fibrotic tissue and the coating of large vessels.

To examine changes in the expression pattern of the lymphatic endothelial cell and mesenchymal markers in acutely and chronically infarcted myocardium, we implemented immunohistochemical analysis of the tissue sections obtained from non-operated mouse hearts, as well as cardiac samples at 4 and 8 hours (< 1 day), 2 days, 2 weeks and 1 month after coronary artery ligation, and sham-operated animals. We observed that at the time of coagulation necrosis and early stages of tissue granulation [12, 24], as illustrated by 8 hours after MI, there was a slight decrease in podoplanin-labeled structures in the necrotic area when compared to a corresponding myocardial region in non-operated hearts (Fig 1A), which is in agreement with previous findings in humans [12]. Unexpectedly, at 2 days after MI, there was a more than a 6-fold rise in the podoplanin immunoreactivity in the infarct BZ relative to earlier time points after MI (< 1 day) or myocardial area remote to infarction (RA) (Figs 1A, 1B, 1E and 2A and S1 Fig Panels A-C and S2 Fig Panels A,B; 2 days).

By flow-cytometry evaluation of isolated cardiac cells, we established that the frequency of podoplanin was relatively low in non-operated and sham-operated non-infarcted hearts (Fig 1C). Of note, a recent study, combining immunohistochemical and flow-cytometry assessments of the mouse cardiac cellular composition, similarly reports that in homeostatic conditions, the podoplanin-positive cells are rare, constituting less than 5% of the myocardial endothelial cell population [23]. We next documented that in line with immunohistochemical findings on the podoplanin accumulation in the infarct BZ, flow-cytometry analysis determined that in the total heart, MI was associated with more than a three-fold increase in the occurrence of podoplanin-expressing cells versus non-operated and sham-operated counterparts (Fig 1C).

In the infarcted myocardium, similarly to remote areas and sham controls, the LYVE-1 labeling coincided with podoplanin almost exclusively in the vessel endothelium (Fig 1E–1H). It is well-documented that lymphangiogenesis in the infarcted heart is peaking with the development of fibrosis and commencement of scar maturation [12–15, 25]. Accordingly, during early inflammatory responses to tissue damage [24], there were no noticeable differences in the presence of LYVE-1-positive CLVs in proximity to the injured area (Fig 1E and S1 Fig; 4 and 8 hours). In contrast, at the time of the appearance of podoplanin-positive cells at 2 days after MI, the co-labeling of LYVE-1 with podoplanin in the infarct BZ was substantially diminished (Fig 1E and 1F and S1 Fig Panels A-E; 2 days). We observed that the expansion of podoplanin-positive compartment shortly after MI was manifested by robust accumulation of interstitial LYVE-1-negative cells in the infarct BZ (Fig 1E and 1F, and S1 Fig Panels A-E; 2 days), and, to a much lesser extent, the RA (Fig 1G and S1 Fig Panels F,G; 2 days). Quantitatively, as compared to RA, in the infarct BZ there was more than a 30-fold decrease in the proportion of podoplanin-positive structures displaying the co-staining with LYVE-1 (Fig 1F, graph; 2 days). In support, flow-cytometry suggested that a much smaller fraction of podoplanin and LYVE-1 double-positive cells resided in the heart after MI compared to non-operated and sham-operated conditions (S3 Fig Panel A). Similarly, the co-labeling with a pan-endothelial determinant PECAM-1 was significantly reduced after MI in the cohorts of podoplanin-presenting cells (S3 Fig Panel B, PECAM-1), further demonstrating that a large share of podoplanin-bearing cells appearing after infarction in the myocardial interstitium did not display markers of mature endothelium.

**Fig 3:**
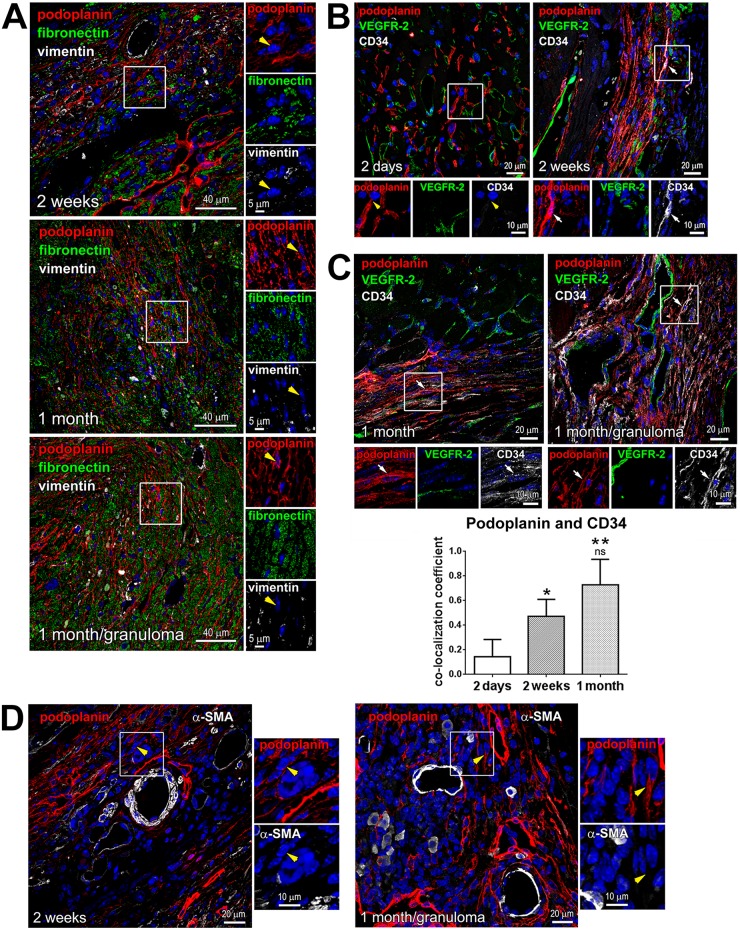
Phenotype of podoplanin-positive cells in the fibrotic tissue. Thin cardiac sections were indirectly immunolabeled with antibodies that recognize podoplanin (red) and either fibronectin and vimentin (**A**; green and grey, respectively), VEGFR-2 and CD34 (**B** and **C**; green and grey, respectively), or α-SMA (**D**; grey). Nuclei, blue. Time after MI is indicated. Areas in rectangles are shown at higher magnifications in the adjacent images for each color channel. Note that vimentin (**A**) or α-SMA (**D**) labeling is rarely detectable in podoplanin-expressing cells (examples are pointed by yellow arrowheads). In **B** and **C**, the podoplanin-presenting cells show minimal VEGFR-2 labeling. At 2 days after MI, the podoplanin-bearing cells mostly do not co-stain with CD34 (exemplified by yellow arrowheads). Starting 2 weeks after MI, the CD34 staining is present in irregular capillary-like structures (examples are indicated by white arrows). In **C**, quantitative image analysis demonstrating changes in the podoplanin co-labeling with CD34 at indicated times after MI is included in the graph (lower panel). Data represent mean and SD of the co-localization coefficient; n = 5–6 image fields per group. By one-way ANOVA, *P < 0.02 for 2 weeks vs. 2 days; **P < 0.0001 for 1 month vs. 2 days; ns, not-significant for 1 month vs. 2 weeks.

**Fig 4:**
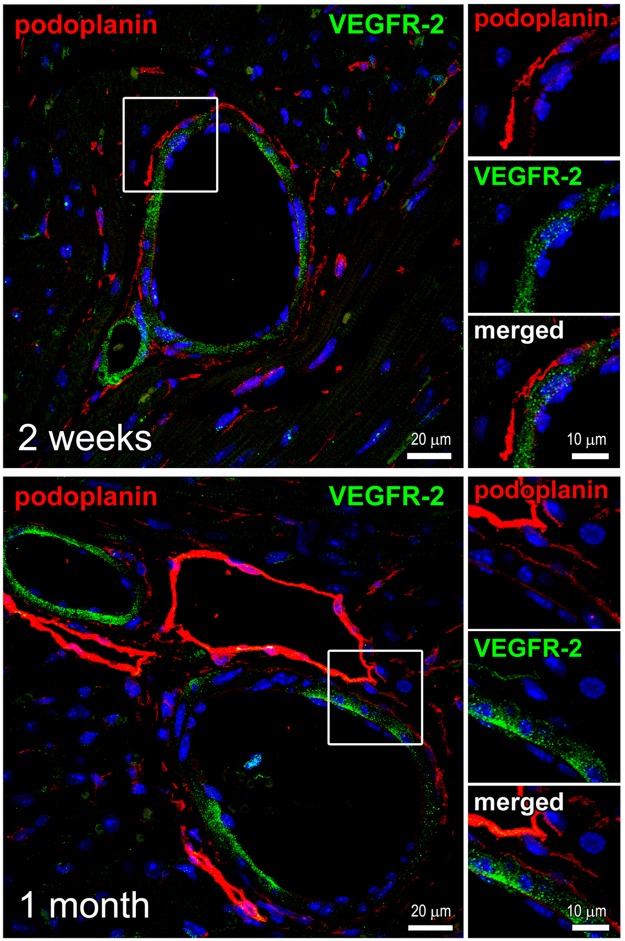
Perivascular localization of the podoplanin-expressing cells. Thin cardiac sections were indirectly immunolabeled with podoplanin (red) and VEGFR-2 (green) antibodies. Nuclei, blue. Time after MI is indicated. Areas in rectangles are shown at a higher magnification in the adjacent images for each color channel and merged. Note the podoplanin-positive cells encircling the VEGFR-2-labeled BVs.

Subsequently, at 2 weeks after MI, at the maturation phase of wound healing, the density of podoplanin-labeled cells and CLVs was further elevated in the scar and BZ: there was an additional 1.7-fold increase in the podoplanin-labeled tissue area relative to 2 days, and a 10-fold rise relative to inflammatory stage (< 1 day) (Fig 1B and 1D–1F and S1 Fig Panels A-E; 2 weeks), or the remote RA (Fig 1B and 1G and S1 Fig Panels F,G) and non-operated myocardium (Figs 1A and 2A and S2 Fig Panels A,B; NO). In the healing scar, podoplanin immunoreactivity was apparent in LYVE-1-negative cell cords (Fig 1E and 1F and S1 Fig Panels A-E; 2 weeks). The proportion of podoplanin-positive cells co-labeled with LYVE-1 at 2 weeks after MI was almost 15 times lower than in the RA (Fig 1F, graph; 2 weeks). These podoplanin-presenting LYVE-1-negative cells were aligned with the extracellular matrix (Fig 3A; 2 weeks) and formed capillary-like structures, which occasionally expressed CD34 (Fig 3B). The co-labeling of podoplanin with CD34 was more readily detectable at the time of scar maturation at 2 weeks compared to an earlier stage of acute injury at 2 days (Fig 3B), with a 3-fold increase in the co-localization coefficient relative to 2 days (Fig 3C, graph). Indeed, as evaluated by flow cytometry, shortly after MI there were no significant changes in the proportion of CD34-positive cells within podoplanin-presenting populations versus sham-operated controls (S3 Fig Panel B, CD34). Intriguingly, the podoplanin-expressing cells also frequently encircled BVs in the fibrotic region and neighboring myocardium (Figs 1F, 3D and 4; 2 weeks), which might point to their origin from perivascular cells or the cells recruited from circulation.

In the heart with a mature scar at 1 month after MI, the podoplanin labeling in the infarcted area and BZ remained high with no significant changes versus 2 weeks after MI (Fig 1B and 1D–1F). As expected [12–16], the presence of LYVE-1- and podoplanin-positive CLVs was prominent (Figs 1D–1F, 1H and 2B, S1 and S2 Figs Panel C; 1 month), supporting the notion that lymphatics, once formed, persist in the scarred tissue [12, 13, 15, 26]. The co-staining with CD34 was also simialr to that in a maturing scar at 2 weeks (Fig 3B; 2 weeks and Fig 3C; 1 month, and Fig 3C, graph), with an almsot 5-fold rise in podoplanin co-localization coefficient with CD34 relative to 2 days after MI (Fig 3C, graph). Correspondingly to the earlier stages of scar maturation, the podoplanin-expressing population in the mature scar was dominated by the LYVE-1-negative multicellular assemblies (Fig 1E and 1F and S1 Fig Panels A-E; 1 month), which were observed aligned with fibronectin deposits in the scar (Fig 3A; 1 month) and at the outline of small and large blood vessels (Fig 4; 1 month). In contrast, there was no such accumulation of the podoplanin-labeled cellular aggregates in the RA not affected by the infarction (Fig 1F, graph, RA, and Fig 1G and S1 Fig Panels F,G; 1 month). Quantitatively, there was more than a 10-fold decrease in the podoplanin and LYVE-1 co-localization in the chronic scar and BZ compared to RA (Fig 1F; 1 month). Collectively, these findings point to a correlation between the presence of podoplanin-expressing LYVE-1-negative cells at different stages of cardiac healing and the development of CLVs and fibrosis after MI.

Of interest, the growth of CLVs and appearance of podoplanin-positive interstitial cells were not detected in the sham-operated animals (Fig 1H and S1 Fig Panel H; 1 month SHAM). Likewise, there were no significant differences in the frequency of podoplanin between the sham- and non-operated hearts at 2 days after surgery (Fig 1C). These data underscore a specific effect of the MI-induced injury on the podoplanin expression and lymphangiogenesis.

### Variable manifestation of the lymphatic endothelial cell markers Prox-1 and VEGFR-3 in the podoplanin-positive cardiac population

**Fig 5:**
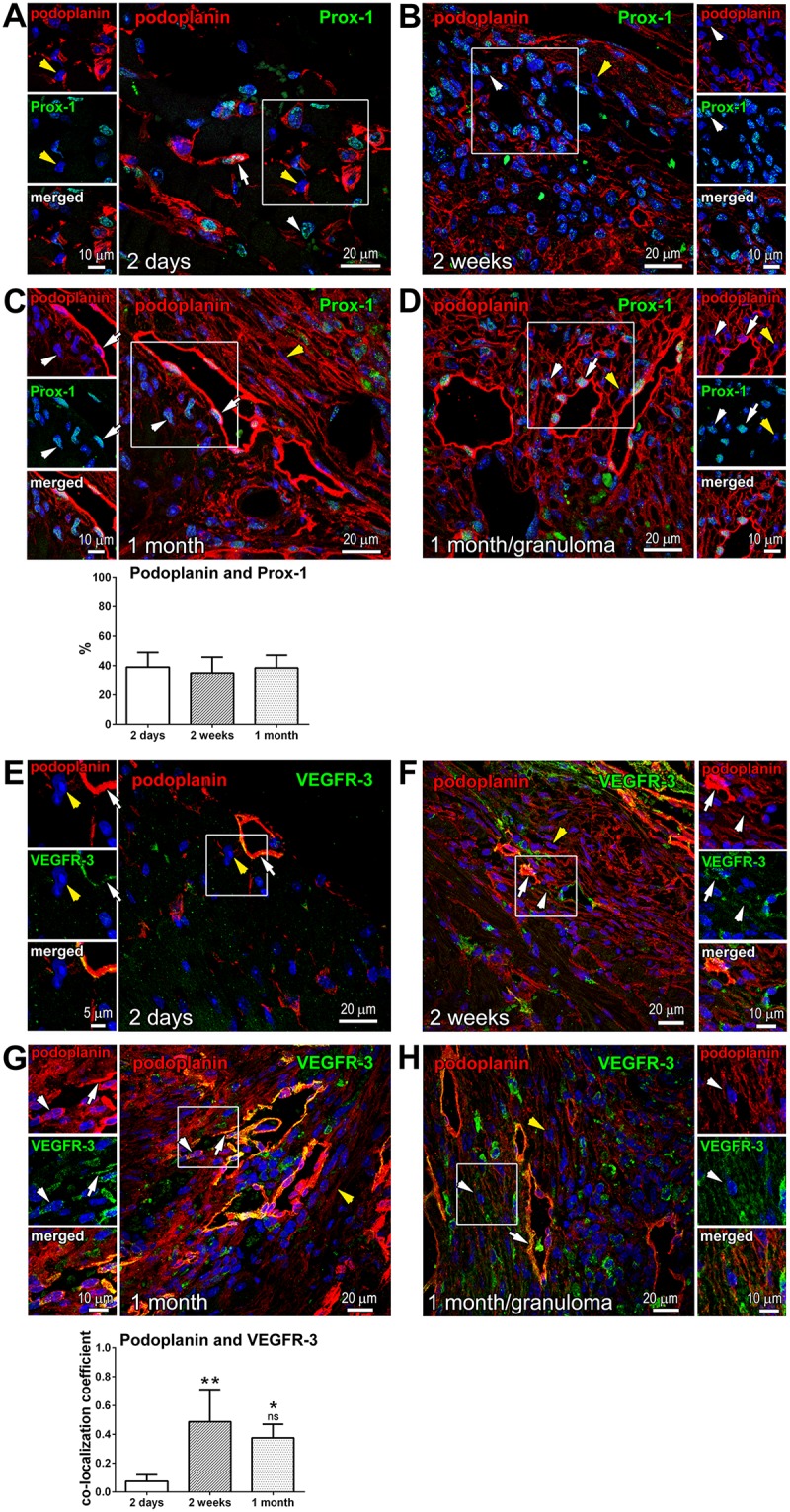
Variable expression of Prox-1 and VEGFR-3 in podoplanin-positive cells in the infarcted heart. Thin cardiac sections were indirectly immunolabeled with antibodies that recognize podoplanin (red) and either Prox-1 (**A**-**D**; green) or VEGFR-3 (**E**-**H**; green). Nuclei, blue. Time after MI is indicated. Areas in rectangles are shown in the adjacent images for each color channel and merged. White arrows indicate examples of Prox-1 or VEGFR-3 labeling in the lymphatic endothelial cells of CLVs, and white arrowheads point to the examples of Prox-1 or VEGFR-3 staining in podoplanin-expressing interstitial cells. Yellow arrowheads exemplify instances of podoplanin-positive cells in which Prox-1 or VEGFR-3 expression was undetectable. Note the consistent detection of Prox-1 and VEGFR-3 in CLVs, and the heterogeneity in Prox-1 and VEGFR-3 labeling intensity in podoplanin-stained cells not organized into vessels. In **C**, quantitative image analysis (graph, lower panel) of the fraction of podoplanin-expressing cells co-labeled with Prox-1 is shown at indicated times after MI. Data represent mean and SD of the % double-positive cells out of all podoplanin-positive cells in the imaging field; n = 6–9 image fields per group. By one-way ANOVA, no significant changes between the groups. In **G**, quantitative image analysis (graph, lower panel) of the podoplanin co-labeling with VEGFR-3 is shown. Data represent mean and SD of the co-localization coefficient measured at indicated times after MI; n = 4–7 image fields per group. By one-way ANOVA, **P = 0.002 for 2 weeks vs. 2 days; *P = 0.009 for 1 month vs. 2 days; ns, not-significant for 1 month vs. 2 weeks.

In order to assess the lymphangiogenic potential of the podoplanin-bearing cells in the infarcted myocardium, we evaluated the presence of a lymphatic endothelial cell-specific transcription factor Prox-1 [27–29] in this population. We noted that at different times after MI, along with the expected localization in the nuclei of the endothelium of CLVs (Fig 5A–5C, arrows), Prox-1 staining was detectable in the podoplanin-positive cells not organized into vessel-like structures (Fig 5A–5C, white arrowheads). By flow-cytometry, the frequency of Prox-1 in podoplanin-positive cells was diminished at 2 days after MI as compared to sham-operated hearts (S3 Fig Panel B, Prox-1). These data are consistent with the findings on a lower presence of LYVE-1 and PECAM-1 in the podoplanin-expressing cell cohorts, thus corroborating the assumption that a large proportion of podoplanin-bearing cells appearing acutely after injury do not possess a differentiated lymphatic endothelial phenotype.

The occurrence of Prox-1 in the infarcted myocardium was generally high at the later stages of wound repair (Fig 5B and 5C; 2 weeks and 1 month). However, there was no direct correlation between the incidences of podoplanin and Prox-1 in these cell cohorts (Fig 5B and 5C, yellow arrowheads). Despite a major rise in the abundance of podoplanin-presenting cells in the scarred tissue (Fig 1B and 1D–1F; 2 days versus 2 weeks and 1 month), we observed that the percentage of podoplanin-labeled Prox-1-postive cells in the injured area remained similar at all the time points after MI (Fig 5C, graph), supporting the notion that many of the newly-appearing podoplanin-expressing cells in the fibrotic area are Prox-1 negative.

VEGFR-3 is another characteristic marker of the lymphatic endothelial cell activation and differentiation [30–32]. In the infarcted myocardium, VEGFR-3 was discontinuously expressed in CLVs (Fig 5E–5G, white arrows), but only occasionally found in the podoplanin-positive cells populating the infarct BZ shortly after MI (Fig 5E and 5G; 2 days). By flow-cytometry, the frequency of VEGFR-3 co-staining with podoplanin was not affected by acute myocardial injury as compared to non-operated and sham-operated animals (S3 Fig Panel B, VEGFR-3). The level of VEGFR-3 immunolabeling was augmented at the later phases of infarct healing at 2 weeks and 1 month, and the co-staining of VEGFR-3 with podoplanin was intensified at these stages (Fig 5F and 5G, white arrowheads). Quantitatively, more than a 5-fold elevation in the co-localization of podoplanin with VEGFR-3 was found at the time of scar formation and maturation relative to 2 days after MI (Fig 5G, graph). Yet, VEGFR-3-expression was frequently lacking in podoplanin-presenting cells (Fig 5F and 5G, yellow arrowheads). The appearance of Prox-1 or VEGFR-3 in a subset of podoplanin-expressing cells suggests their commitment to the lymphatic endothelial cell fate. At the same time, the absence of lymphendothelial epitopes in a large group of podoplanin-positive cells might signify an alternative differentiation pathway.

In contrast to VEGFR-3, the presence of VEGFR-2 was rarely detected in podoplanin-bearing interstitial cells and CLVs in the infarcted heart (Figs 3B and 3C and 4). This is in agreement with previous reports demonstrating that in the mouse, VEGFR-2 is restricted to the activated blood endothelium [32].

### Temporal changes in the mesenchymal markers PDGFRα and PDGFRβ in podoplanin-positive cardiac cells

**Fig 6:**
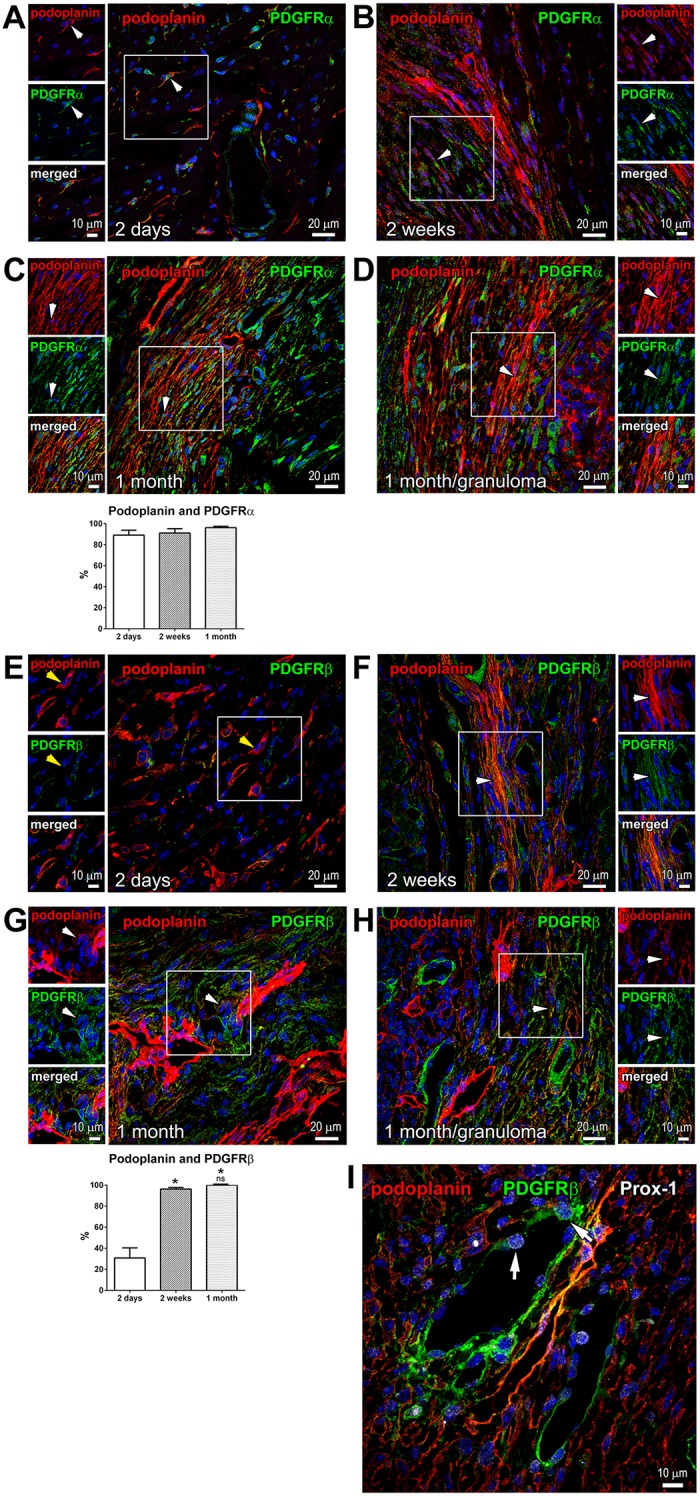
Expression of PDGFRα and PDGFRβ in podoplanin-positive populations. Thin cardiac sections were indirectly immunolabeled with podoplanin (red) and either PDGFRα (**A**-**D**; green), PDGFRβ (**E**-**H**; green), or PDGFRβ and Prox-1 (**I**; green and grey, respectively) antibodies. Nuclei, blue. In **A**-**H**, time after MI is indicated. Areas in rectangles are shown in the adjacent images for each color channel and merged. In **A**-**D**, podoplanin is frequently co-stained with PDGFRα at every time point; examples are indicated by white arrowheads. In **E**, podoplanin-positive cells are mostly PDGFRβ-negative, as exemplified by yellow arrowheads. In **F**-**H**, PDGFRβ distinctly co-stained with podoplanin. In **C** and **G**, quantitative image analyses (graphs, lower panels) of the fraction of podoplanin-expressing cells co-labeled with PDGFRα (**C**) or PDGFRβ (**G**) are shown at indicated times after MI. Data represent mean and SD of the % double-positive cells out of total podoplanin-positive cells; n = 5 image fields per group. By one-way ANOVA for PDGFRβ, *P < 0.0001 for 2 days vs. 2 weeks or 1 month; ns, not-significant for 1 month vs. 2 weeks. In **I**, arrows point to the examples of Prox-1 staining in the nuclei of PDGFRβ-positive podoplanin-negative BVs.

In the infarcted myocardium, the heightened expression of PDGFRα, PDGFRβ and their PDGF ligands coincides with angiogenesis and inflammatory and fibrogenic responses, indicating a role in wound repair processes [33, 34]. We found by flow-cytometry analysis of cardiac cells from acutely infarcted hearts (S4 Fig), and immunohistochemistry assessment of myocardium at the different times after MI (Fig 6A–6C, white arrowheads, and Fig 6C graph), that the podoplanin-presenting cells in the infarcted heart were distinctly PDGFRα-positive. Since PDGFRα expression is associated with the properties of immature mesenchymal cells [35–39], the concordance of PDGFRα and podoplanin staining suggests that cardiac podoplanin-positive cells contain a population with progenitor cell capabilities.

Unlike PDGFRα, PDGFRβ was infrequent in the podoplanin-presenting cells early after MI (Fig 6E; 2 days, yellow arrowheads, Fig 6G, graph; 2 days, and S4 Fig). However, we noticed that the level of PDGFRβ expression and co-staining with podoplanin were strongly elevated at the later stages of infarct healing and in the mature scar (Fig 6F and 6G; 2 weeks and 1 month, white arrowheads), reaching ~100% co-labeling of the podoplanin-positive cells with PDGFRβ (Fig 6G, graph; 2 weeks and 1 month).

PDGFRβ is a marker of pericytes and perivascular cells with fibrogenic potential [40–42]. In the infarcted myocardium, PDGFRβ labeling was abundant in the CLVs and BVs (Fig 6I). Surprisingly, we noticed Prox-1 expression in the nuclei of podoplanin-negative cells of the BVs (Fig 6I, arrows). Interestingly, a recent lineage-tracing analysis shows that PDGFRβ-positive endothelium contributes to the CLV formation during embryonal development [14], implying that upon severe tissue injury, blood endothelial cells acquire the lymphatic endothelial cell phenotype. Such endothelial cell plasticity has been previously described in cultured cells and for the tumor vasculature [43–45].

Of note, although the expression of PDGFR α or β is often related to the fibrogenic behavior and associated with the myofibroblast phenotype of cells [35–38, 40], we found that the podoplanin-positive populations in the scar seldom exhibited fibroblast markers vimentin (Fig 3A, yellow arrowheads) or a myofibroblast protein α-SMA (Fig 3D, yellow arrowheads). This suggests that the podoplanin-labeled cells in the heart do not generate fully-differentiated fibroblasts, or the podoplanin expression is lost in the maturing fibrogenic cells.

### Evidence for the role of inflammation in the recruitment of podoplanin-expressing cells

Inflammation-induced lymphangiogenesis is a well-established phenomenon implicated in wound healing responses [26, 46, 47]. Granuloma is a form of inflammatory reaction described for several diseases. It is noted that nodules of granulomas in different tissues are characterized by the presence of podoplanin-positive cells and lymphatic vessels of heterogeneous and atypical morphology, which frequently express PDGFRβ [42, 48, 49], resembling the podoplanin-labeled cells in the chronically infarcted heart (Fig 6G; 1 month).

In the present study we also examined few cases of granulomas that developed near the insertion of a suture thread within the myocardium. We found a high density of podoplanin labeling in the granuloma nodules in the heart (Fig 1D–1F, S1 Fig Panels A-E and Fig 3A and 3C; 1 month/granuloma), but not in the RA of the same samples (Fig 1G and S1 Fig Panels F,G; 1 month/granuloma). Additionally, the frequency of podoplanin-positive cells that express Prox-1 or VEGFR-3 was increased in the scar and BZ in the cardiac samples with MI and granulomas as compared to MI only (Fig 5C and 5G; 1 month and Fig 5D and 5H; 1month/granuloma). Although the lymphangiogenic responses in the infarcted myocardium with granulomas were apparently amplified relative to MI only (Fig 1D), the phenotypic features of podoplanin-labeled cells in the hearts with granulomas, including vascular markers (Fig 3C; 1 month/granuloma) and PDGFRα and PDGFRβ (Fig 6D and 6H; 1month/granuloma), were similar to the ones observed in the absence of granuloma (Figs 3C and 6C and 6G; 1 month). This suggests that the surge in podoplanin expression following acute MI is at least partly driven by inflammation.

**Fig 7:**
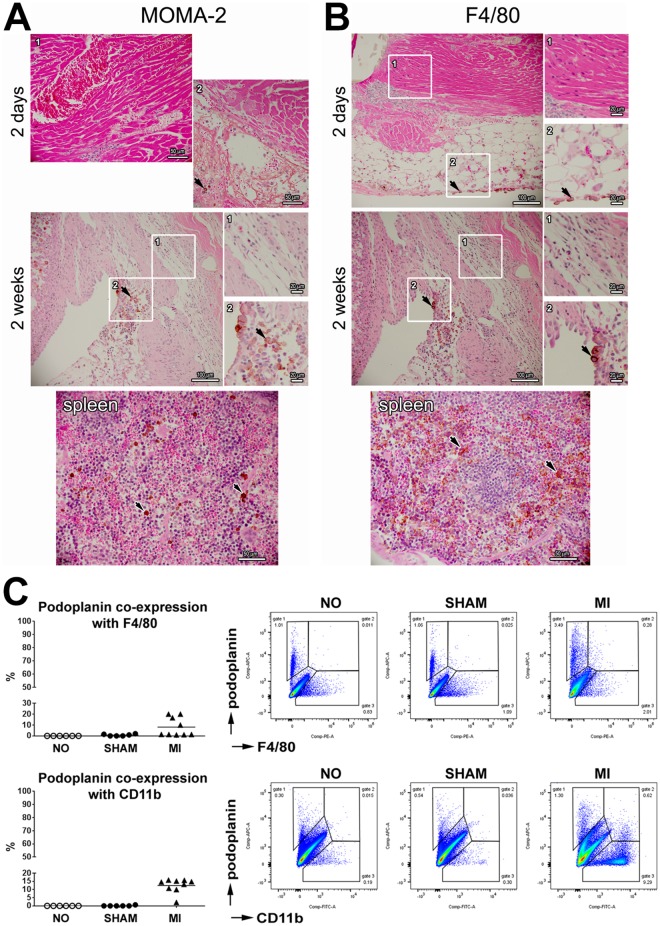
Low expression of monocyte-macrophage markers in the populations of podoplanin-positive cells in the infarcted myocardium. (**A**,**B**) Thin cardiac sections were indirectly immunolabeled with MOMA-2 (**A**) or F4/80 (**B**) antibodies and counterstained with hematoxylin and eosin. Time after MI is indicated. Spleen sections were included as positive controls. Areas in rectangles with the corresponding numbers are shown at a higher magnification in the adjacent insets. Note the presence of cells immunoreactive for MOMA-2 or F4/80 (arrows) in the inflamed epicardium (2 days, insets 2), myocardial interstitium (2 weeks, insets 2) and spleen, but not in the BZ of necrotic myocardium (2 days, insets 1) or the maturing scar (2 weeks, insets 1). (**C**) Flow-cytometry analysis of the F4/80 or CD11b expression in the podoplanin-positive cohorts populating the hearts of non-infarcted (NO), sham-operated (SHAM) and infarcted (MI) mice at 2 days after surgery. Graphs displaying individual values and the respective means (left), as well as representative scatterplots (right) are shown. Data represent frequency of double-positive cells within podoplanin-labeled populations (calculated as % cells in gate 2 out of the sum of cells in gate 1 and gate 2).

The inflammatory reaction to myocardial injury is evident by a time-dependent accumulation of immune effectors, including macrophages. In murine embryos and pathological conditions, cells that exhibit traits of macrophages display characteristics of lymphatic endothelial cells and localize in the regions of lymphatic vessel growth, serving as a source of lymphangiogenic factors, and potentially integrating into newly-formed vessels [50–53]. We did not detect the presence of monocyte-macrophage markers MOMA-2 (Fig 7A), F4/80 (Fig 7B), or CD11b and Ly6C (S5 Fig) in the infarcted myocardial areas that were characterized by the accumulation of podoplanin-positive cells (Fig 1). Furthermore, by flow-cytometry analysis we documented that at 2 days after MI on average only 20% of podoplanin-positive cells in the infarcted heart were labeled with either F4/80, CD11b (Fig 7C) or Ly6C (S5 Fig) antibodies. These data indicate that the podoplanin-expressing pools in the heart in majority do not correspond to maturing macrophages, although, as discussed below, their hematopoietic origin cannot be excluded [54].

## Discussion

In the present work we studied potential cellular mediators of the lymphangiogenic and fibrogenic responses associated with the different stages of myocardial wound repair after infarction.

We established that shortly after MI, at the time corresponding to the later phases of granulation of the necrotic tissue in the infarcted myocardium, there was more than a six-fold increase in the podoplanin labeling at the areas neighboring the infarction as compared to remote area, and a three-fold increase in the frequency of podoplanin-positive cells in the whole heart relative to non-operated and sham-operated controls. These newly-appearing podoplanin-presenting cells not organized into vessel-like structures were predominantly LYVE-1-negative and exhibited a heterogeneous phenotype in terms of various markers of mesenchymal and endothelial fates. The immunolabeling profile of podoplanin-expressing cells at the different stages of infarct repair is summarized in Table 1.

**Table 1:** Summary of the observed immunolabeling profile of podoplanin-expressing cells at different stages of infarct repair ^(a)^^,^^(b)^^,^^(c)^^,^^(d)^.

Time after MI	2 days			2 weeks			1 month		
*Myocardial region*	*BZ*^(§)^	*BZ*	*RA*^(^*^)^	*scar and BZ*	*scar and BZ*	*RA*^(^*^)^	*scar and BZ*	*scar and BZ*	*RA*^(^*^)^
*Relative location of podoplanin-positive cells*	^(1)^interst. and perivas.	^(2)^lymph. vessels	^(2)^lymph. vessels	^(1)^interst. and perivas.	^(2)^lymph. vessels	^(2)^lymph. vessels	^(1)^interst. and perivas	^(2)^lymph. vessels	^(2)^lymph. vessels
*Podoplanin co-labeling with*:									
**LYVE-1**	-	+	++	-	+	++	-	+	++
**Prox-1**	-/+	++	+	-/+	++	+	-/+	++	+
**VEGFR-3**	-	+	+	-/+	+	+	-/+	+	+
**CD34**	-	-	-	-/+	-/+	-/+	+	-/+	-/+
**PDGFRα**	++	++	-/+	++	++	-/+	++	++	-/+
**PDGFRβ**	-	-/+	-	++	++	-	++	++	-
**α-SMA**	-	-	-	-	-	-	-	-	-

^(a)^ - designates that < 20% cells are co-labeled

^(b)^ -/+ designates that 20–50% cells are co-labeled

^(c)^ + designates that > 50% cells are co-labeled

^(d)^ ++ designates that all the cells are co-labeled

^(§)^ no scar was formed at 2 days

^(^*^)^ interstitial and perivascular cells were seldom detected in the RA

^(1)^ interst. and perivas. designates interstitial and perivascular cells; comparisons pertaining to interstitial and perivascular podoplanin-positive cells are shaded

^(2)^ lymph. vessels designates cells organized in lymphatic vessels

The presence of podoplanin-bearing cells was mainly prominent in the areas of future lymphangiogenesis at the infarct BZ. The abundance of podoplanin was further elevated almost two-fold in the healing and maturing scars but not the RA, concomitantly with the previously reported increase in LYVE-1 and VEGFR-3-positive CLVs [12–16] and the buildup of fibronectin deposits [24–25]. The expansion of podoplanin-positive compartment and CLVs was not noticeable in the myocardium of sham-operated animals.

We also found that the accumulation of podoplanin-labeled multicellular assemblies was intensified in vicinity of myocardial granuloma nodules, exhibiting similarities to encapsulating peritoneal sclerosis [48] and pulmonary sarcoid granulomas [49]. Therefore, inflammatory processes might play a significant role in the recruitment of podoplanin-bearing LYVE-1-negative cells to the site of myocardial repair or the activation of podoplanin expression in responsive cell cohorts. Indeed, homing of circulating cells is proposed to contribute to the lymphatic vessel formation under inflammatory conditions [54–56], with evidence that rare bone marrow-derived podoplanin-positive cells express Prox-1 and function as lymphatic endothelial progenitors [56]. In addition, inflammation and neoplastic growth alter the podoplanin level in various cell types, impacting their differentiation status and migratory behavior [57, 58].

Although the podoplanin-mediated signaling pathway is not sufficiently understood, the expression of podoplanin is cognate to lymphatic endothelial cells; and inflammatory lymphangiogenesis is attenuated if podoplanin activity is lacking [59–61]. Podoplanin deficiency impairs cardiac development [59], while continuous expression of podoplanin into adulthood is required to maintain functional lymphatic vasculature [57, 62, 63]. Accordingly, we detected the presence of canonical lymphendothelial markers Prox-1 and VEGFR-3 within the cohort of podoplanin-positive LYVE-1-negative cells (Table 1), which might be indicative of their differentiation into lymphatic endothelial cells of CLVs. Additionally, a hematoendothelial epitope CD34 was observed in podoplanin-presenting cells, and its expression was augmented with time after MI (Table 1). CD34 labeling may signify a hematopoietic origin of these cells. Moreover, CD34 upregulation distinguishes lymphatic endothelium in tumors [64]. Hence the presence of CD34 in podoplanin-expressing cells and vessels of the chronically infarcted heart might point to their activated diseased state.

Nevertheless, a considerable fraction of the podoplanin-presenting cells, which was seemingly indistinguishable from the above cell group in terms of morphology and tissue location, did not exhibit the markers associated with endothelial commitment, namely Prox-1, VEGFR-3, CD34 (Table 1). These differences might be caused by a transient nature of expression of the factors governing the growth and differentiation of lymphatic endothelium [65]. Alternatively, our findings imply that podoplanin-positive compartment in the infarcted myocardium constitutes an inhomogeneous population, consisting of cells with a variable potency to adopt the lymphatic endothelial or other cell fates. For instance, it has been documented that interstitial stroma cells acquire podoplanin expression after organ injury and in pathological conditions accompanied by fibrosis in the skin, skeletal muscle and peritoneum [35, 48, 66].

Furthermore, we documented for the first time that a mesenchymal marker PDGFRα was highly represented in podoplanin-positive cells in the wounded heart (Table 1). PDGFRα-expressing cells are proposed to function as mesenchymal progenitors, which in response to injury and inflammation reveal plasticity regarding their ability to differentiate into endothelium or act as profibrotic cells [35, 36, 38, 40]. The predisposition towards the fibrogenic phenotype is influenced by the presence of pathologies and with aging, and can be antagonized to reduce scarring and improve angiogenesis [67]. Thus, the high expression of PDGFRα, along with irregular occurrences of Prox-1 and VEGFR-3 (Table 1), might signify an alternative ability of podoplanin-bearing cells to generate lymphatic endothelial cells or fibroblasts, impacting the outcome of the myocardial repair process. Likewise, in the human liver, podoplanin discriminates disparate categories of progenitor and stromal cell subsets, which display cell fate plasticity, population growth, and alterations in the relative distribution under conditions of chronic inflammation and fibrosis [68].

PDGFRβ is also linked to the fibrogenic activity of mural cells [40, 41]. Inflamed and fibrotic tissues are characterized by the abundance of podoplanin-presenting cells of mesenchymal morphology, which often co-express PDGFRβ [42, 48, 49]. Of interest, granuloma nodules in the heart showed similar accumulation of podoplanin and PDGFRβ co-presenting cells. In the infarcted myocardium with no granulomas, the high occurrence of PDGFRβ in podoplanin-bearing cells was apparent as well, albeit at the later stages of wound repair, concomitantly with the scar development (Table 1). Disruption of the PDGFRβ signaling impairs post-MI angiogenesis and BV maturation, and decreases collagen content in the wound, destabilizing the scar [69]. Therefore, the acquisition of PDGFRβ by podoplanin-positive cells in the chronically infarcted myocardium might reflect their active role in lymphangiogenesis, fibrotic responses and scar maintenance.

Yet, the markers of fibroblast, such as α-SMA, were rarely detected in podoplanin-positive cells residing in the fibrotic areas (Table 1). Although this observation does not exclude the fibrogenic potential of the podoplanin-presenting population in the infarcted heart, it suggests a lack of full transformation into myofibroblasts. Analogous type of PDGFRα-positive progenitors, which acquire fibrogenic behavior due to a partial endothelial-mesenchymal transition but do not become myofibroblasts, has been reported in the injured muscle [35, 38].

The growth of lymphatic network in adult organs is believed to occur as a result of the proliferative expansion and/or sprouting of new lymphatic vessels from pre-existing lymphatics [26, 46, 70]. These processes are seemingly conditioned by the type of stimulus: whereas VEGFR-2 activation mainly induces vessel enlargement, VEGFR-3 signaling promotes sprouting [30, 31]. Notably, VEGFRs, as well as VEGF-C and -D ligands, are up-regulated in the peri-infarcted region [12, 14, 15, 71]. Administration of a selective VEGFR-3 agonist to the infarcted heart induces strong and sustained lymphangiogenesis [14, 15]. VEGFR-3 signaling is enhanced by mechanical stretch [72], which may explain the development of the lymphatic vasculature when interstitial pressure is elevated. Intriguingly, activation of podoplanin on the lymph node reticular cells diminishes their contractility, altering organ shape and stiffness under inflammatory conditions [73]. Thus, podoplanin-positive cells in the infarcted myocardium might affect local tissue tension, indirectly impacting VEGFR-3-stimulated CLV growth, extracellular matrix deposition and scar remodeling.

In conclusion, our detailed spatiotemporal analysis of the acutely and chronically infarcted myocardium shows that podoplanin expression in the heart identifies structurally and phenotypically diverse cell categories, displaying epitopes of fibrogenic and endothelial commitment. Further studies are warranted to determine whether cells with lymphangiogenic or profibrotic potentials can be recognized within the heterogeneous podoplanin-presenting populations and utilized to promote the CLV growth and attenuate the development of fibrosis after MI.

## Supporting information

S1 Table Primary and secondary reagents employed for immunolabeling, light microscopy, and flow-cytometry. (PDF)

S1 Fig Single channel images composing the merged images presented in the main text Fig 1. Nuclei, blue. (**A**-**C**) Immunolabeling of podoplanin (**A**; red), LYVE-1 (**B**; green) and PECAM-1 (**C**; grey) included in Fig 1E. (**D**,**E**) Immunolabeling of podoplanin (**D**; red) and LYVE-1 (**E**; green) included in Fig 1F. (**F**,**G**) Immunolabeling of podoplanin (**F**; red) and LYVE-1 (**G**; green) included in Fig 1G. (**H**) Immunolabeling of podoplanin (red; upper row), LYVE-1 (green; middle row) and PECAM-1 (grey; lower row) included in Fig 1H.
(PDF)

S2 Fig Single channel images composing the merged images presented in the main text Fig 2. (**A**,**B**) Immunolabeling of LYVE-1 and podoplanin (**A**; red) and isolectin GS-IB4 (**B**; green) included Fig 2A. (**C**) Immunolabeling of LYVE-1 and podoplanin (red; upper row), isolectin GS-IB4 (green; middle row) and α-SMA (grey; lower row) included in Fig 2B.
(PDF)

S3 Fig Flow-cytometry analysis of the podoplanin co-expression with LYVE-1 in cardiac cells from non-operated (NO), sham-operated (SHAM) and infarcted (MI) mice at 2 days after surgery. (**A**,**B**) Isolated cardiac cells were co-stained with podoplanin and the indicated antibodies. (**A**) LYVE-1. Representative scatterplots are shown. Numbers indicate proportions of LYVE-1-positive cells within total podoplanin-positive populations (calculated as % cells in Q2 out of the sum of Q1 and Q2). Samples labeled with non-immune IgGs (IgGs) and podoplanin only or LYVE-1 only were used to determine the gates and calculate background. (**B**) Frequency (%) of podoplanin-positive cells co-labeled with PECAM-1, CD34, Prox-1, or VEGFR-3, respectively, was calculated as in (A). Graphs displaying values for the individual hearts and the respective means for each group (red lines) are shown. **PECAM-1**: ns, not significant; *P = 0.0001 by one-way ANOVA. **CD34**: ns, not significant for SHAM vs. MI by two-tailed *t*-test. **Prox-1**: *P = 0.0036 for SHAM vs. MI by two-tailed *t*-test. **VEGFR-3**: ns, not significant by one-way ANOVA.
(PDF)

S4 Fig Flow-cytometry analysis of the podoplanin co-expression with PDGFRα and PDGFRβ in cells from the infarcted (MI) hearts at 2 days after surgery. Representative scatterplots (left) and the summary graph (right) displaying individual values for each heart with the respective means of the frequency of podoplanin-positive cells co-labeled with PDGFRα or PDGFRβ are shown. Calculated as % cells in gate Q6 out of the sum of the gates Q5 and Q6. Samples labeled with non-immune IgGs (IgGs) and podoplanin only, or PDGFRα or PDGFRβ only (PDGFR only), were used to determine the gates and calculate background.
(PDF)

S5 Fig Low expression of myelo-monocytic markers in the podoplanin-positive interstitial cells. (**A**) Thin cardiac sections 1 month after MI were indirectly immunolabeled with podoplanin (red) and a combination of CD11b and Ly6C (green) antibodies. Nuclei, blue. Area in rectangle is shown at a higher magnification in the adjacent images for each color channel and merged. (**B**) Flow-cytometry analysis of the podoplanin co-expression with Ly6C in cardiac cells from the infarcted (MI) mice at 2 days after surgery. Isolated cells were co-stained with podoplanin and Ly6C antibodies. Representative scatterplots (left) and the graphs displaying individual values with the respective mean (right) are shown. Samples labeled with non-immune IgGs (IgGs) and podoplanin only or Ly6C only were used to determine the gates and calculate the background. Data showing frequencies of double-positive cells within podoplanin-labeled populations in each heart (n = 4) was calculated as % cells in gate 2 out of the sum of % cells in gate 1 and gate 2.
(PDF)
