# Supplementary material for: Phenotypically heterogeneous podoplanin-expressing cell populations are associated with the lymphatic vessel growth and fibrogenic responses in the acutely and chronically infarcted myocardium
Source: PLoS One. 2017 Mar 23;12(3):e0173927. doi: 10.1371/journal.pone.0173927 (PMC5363820; doi:10.1371/journal.pone.0173927)
Supplement: S1 Table — (PDF) [file pone.0173927.s001.pdf]

Supporting Information

S1 Table. Primary and secondary reagents employed for immunolabeling, light microscopy, and flow-cytometry

**Primary Antibodies**				
Antigen	***Manufacturer***	***Catalogue number***	***Host species***	***Applications***
podoplanin	RnD Systems	AF3244	goat	IHC(*), FC(*)
podoplanin	Thermo Fisher Scientific	MA5-1613	syrian hamster	IHC(*)
APC(§)-podoplanin	BioLegend	127409	syrian hamster	FC(*)
LYVE-1	RnD Systems	AF2125	goat	IHC(*)
LYVE-1	Abcam	ab14917	rabbit	IHC(*), FC(*)
PECAM-1	RnD Systems	AF3628	goat	IHC(*)
PECAM-1	BD Pharmingen	553370	rat	FC(*)
Prox-1	Abcam	ab37128	rabbit	IHC(*),FC(*)
Prox-1	Millipore	AB5475	rabbit	FC(*)
PDGFRα	Cell Signaling Technology	3174	rabbit	IHC(*)
PE(§)-PDGFRα	BioLegend	135905	rat	FC(*)
PDGFRα	RnD Systems	AF1062	goat	FC(*)
PDGFRβ	Cell Signaling Technology	3169	rabbit	IHC(*)
PDGFRβ	Abcam	ab51876	rat	IHC(*)
PE(§)-PDGFRβ	BioLegend	136005	rat	FC(*)
VEGFR-3	BD Biosciences	552857	rat	IHC(*)
PE(§)-VEGFR-3	Miltenyi Biotech	130-102-216	rat	FC(*)
VEGFR-2	Cell Signaling Technology	9698	rabbit	IHC(*)
CD34	BioLegend	119301	rat	IHC(*)
PE(§)-CD34	BD Pharmingen	551387	rat	FC(*)

vimentin	Sigma-Aldrich	V2258	mouse	IHC(*)
fibronectin	Abcam	ab2413	rabbit	IHC(*)
α-SMA	Sigma-Aldrich	A2547	mouse	IHC(*)
MOMA-2	AbD Serotec	MCA519G	rat	IHC(*)
F4/80	AbD Serotec	MCA497GA	rat	IHC(*)
PE(§)-F4/80	BioLegend	123109	rat	FC(*)
CD11b	AbD Serotec	MCA74GA	rat	IHC(*), FC(*)
Ly6C	AbD Serotec	MCA2389T	rat	IHC(*)
Alexa Fluor 647-Ly6C	BioLegend	128009	rat	FC(*)
**Non-immune IgGs**				
Description	***Manufacturer***	***Catalogue number***	***Host species***	***Applications***
Normal IgG control	RnD Systems	AB-108-C	goat	FC(*)
Normal IgG control	RnD Systems	AB-105-C	rabbit	FC(*)
APC(§)-IgG control	BioLegend	402012	syrian hamster	FC(*)
PE(§)-IgG2a control	BioLegend	400507	rat	FC(*)
PE(§)-IgG2a control	BD Pharmingen	553930	rat	FC(*)
IgG2a control	BioLegend	400501	rat	FC(*)
APC(§)-IgG2a control	eBioscience	17-432181	rat	FC(*)
**Secondary Antibodies**				
Reactivity	***Manufacturer***	***Catalogue number***	***Conjugate***	***Applications***
Goat	Jackson ImmunoResearch	705-095-147	FITC(§)	IHC(*), FC(*)
Goat	Jackson ImmunoResearch	705-025-147	TRITC(§)	IHC(*)
Goat	Jackson ImmunoResearch	705-175-147	Cy(§)5	IHC(*), FC(*)
Goat	Jackson ImmunoResearch	705-605-147	Alexa Fluor 647	IHC(*), FC(*)
Goat	Jackson ImmunoResearch	705-475-003	DyLight 405	IHC(*)
Hamster	Jackson ImmunoResearch	107-166-142	Cy(§)3	IHC(*)

Hamster	Jackson ImmunoResearch	107-606-142	Cy(§)5	IHC(*)
Hamster	Jackson ImmunoResearch	107-475-142	DyLight 405	IHC(*)
Rabbit	Life Technologies	A21206	Alexa Fluor 488	IHC(*), FC(*)
Rabbit	Life Technologies	A10042	Alexa Fluor 568	IHC(*)
Rabbit	Jackson ImmunoResearch	711-605-152	Alexa Fluor 647	IHC(*), FC(*)
Rabbit	eBioscience	12-4739-81	PE(§)	FC(*)
Rabbit	Jackson ImmunoResearch	711-475-152	DyLight 405	IHC(*)
Mouse	Jackson ImmunoResearch	715-025-140	TRITC(§)	IHC(*)
Mouse	Jackson ImmunoResearch	715-605-140	Cy(§)5	IHC(*)
Mouse	Jackson ImmunoResearch	715-095-150	FITC(§)	IHC(*)
Mouse	Jackson ImmunoResearch	715-025-150	TRITC(§)	IHC(*)
Mouse	Jackson ImmunoResearch	715-175-150	Cy(§)5	IHC(*)
Mouse	Jackson ImmunoResearch	715-475-150	DyLight 405	IHC(*)
Rat	Jackson ImmunoResearch	712-095-150	FITC(§)	IHC(*), FC(*)
Rat	Jackson ImmunoResearch	712-025-150	TRITC(§)	IHC(*)
Rat	Vector	SK-4100	HRP(§)	IHC(*)

(*) IHC= immunohistochemistry; FC= flow cytometry

(§) APC=allophycocyanin; PE= phycoerythrin; FITC= fluorescein isothiocyanate; TRITC= tetramethylrhodamine; Cy= Cyanine; HRP= Horseradish peroxidase
