# Supplementary figures and images for: Phenotypically heterogeneous podoplanin-expressing cell populations are associated with the lymphatic vessel growth and fibrogenic responses in the acutely and chronically infarcted myocardium
Source: PLoS One. 2017 Mar 23;12(3):e0173927. doi: 10.1371/journal.pone.0173927 (PMC5363820; doi:10.1371/journal.pone.0173927)

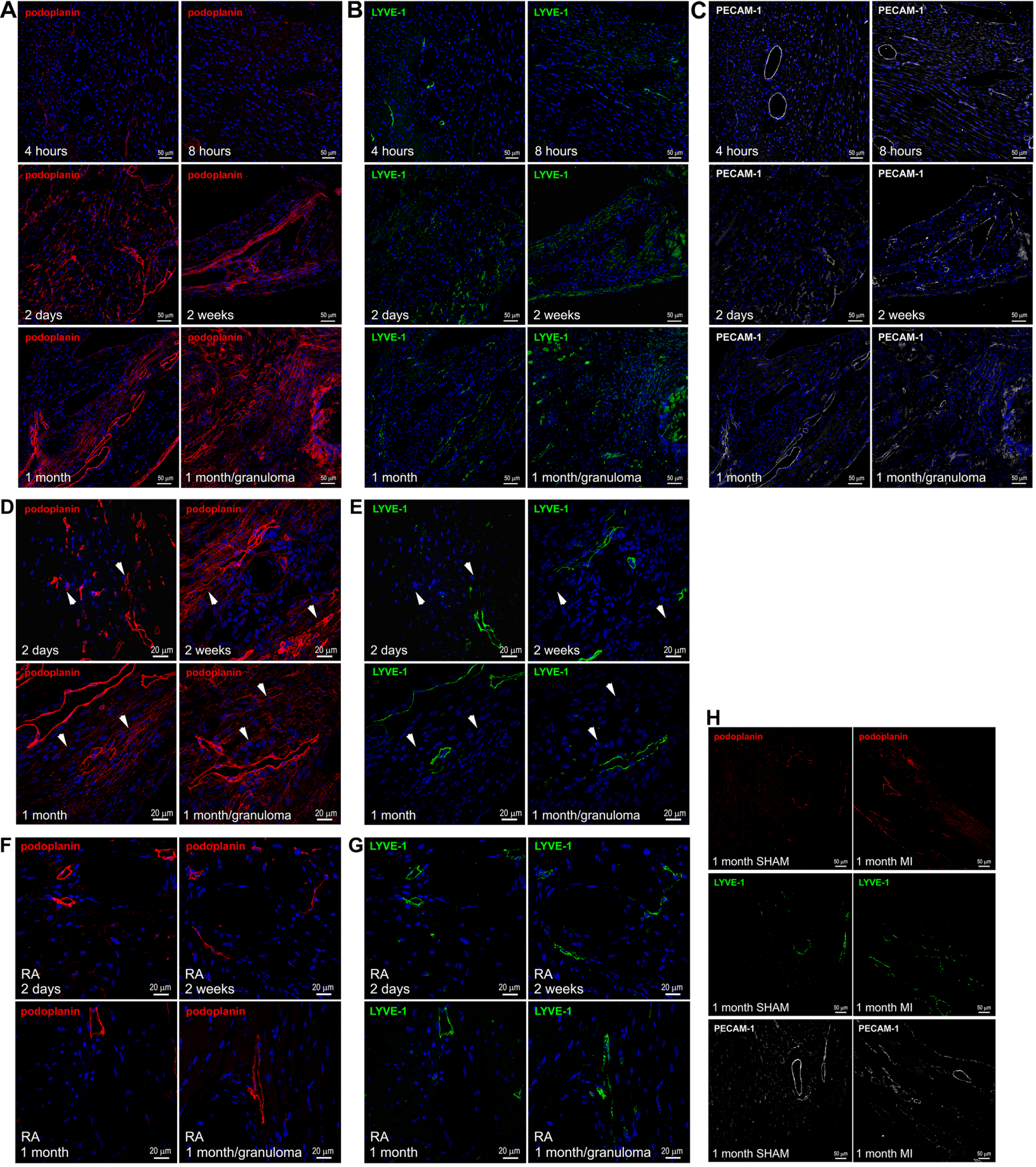

Supplement: S1 Fig — Nuclei, blue. (A-C) Immunolabeling of podoplanin (A; red), LYVE-1 (B; green) and PECAM-1 (C; grey) included in Fig 1E. (D,E) Immunolabeling of podoplanin (D; red) and LYVE-1 (E; green) included in Fig 1F. (F,G) Immunolabeling of podoplanin (F; red) and LYVE-1 (G; green) included in Fig 1G. (H) Immunolabeling of podoplanin (red; upper row), LYVE-1 (green; middle row) and PECAM-1 (grey; lower row) included in Fig 1H. (PDF) [file pone.0173927.s002.pdf]

**A**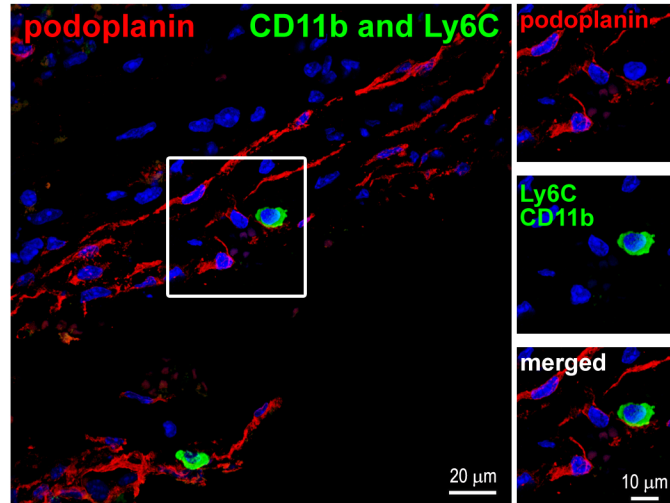**B**

podoplanin  
↑  
→ Ly6C

**IgGs****podoplanin only****Ly6C only****MI****Podoplanin co-expression  
with Ly6C**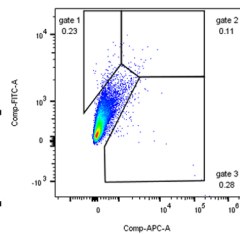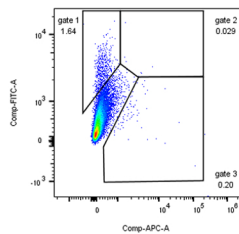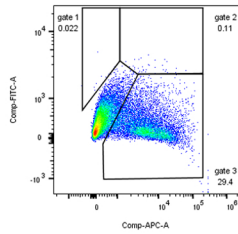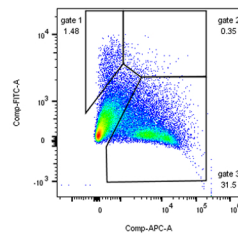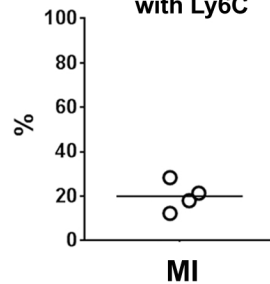

Supplement: S5 Fig — (A) Thin cardiac sections 1 month after MI were indirectly immunolabeled with podoplanin (red) and a combination of CD11b and Ly6C (green) antibodies. Nuclei, blue. Area in rectangle is shown at a higher magnification in the adjacent images for each color channel and merged. (B) Flow-cytometry analysis of the podoplanin co-expression with Ly6C in cardiac cells from the infarcted (MI) mice at 2 days after surgery. Isolated cells were co-stained with podoplanin and Ly6C antibodies. Representative scatterplots (left) and the graphs displaying individual values with the respective mean (right) are shown. Samples labeled with non-immune IgGs (IgGs) and podoplanin only or Ly6C only were used to determine the gates and calculate the background. Data showing frequencies of double-positive cells within podoplanin-labeled populations in each heart (n = 4) was calculated as % cells in gate 2 out of the sum of % cells in gate 1 and gate 2. (PDF) [file pone.0173927.s006.pdf]
