# Supplementary material for: Phenotypically heterogeneous podoplanin-expressing cell populations are associated with the lymphatic vessel growth and fibrogenic responses in the acutely and chronically infarcted myocardium
Source: PLoS One. 2017 Mar 23;12(3):e0173927. doi: 10.1371/journal.pone.0173927 (PMC5363820; doi:10.1371/journal.pone.0173927)
Supplement: S2 Fig — (A,B) Immunolabeling of LYVE-1 and podoplanin (A; red) and isolectin GS-IB4 (B; green) included Fig 2A. (C) Immunolabeling of LYVE-1 and podoplanin (red; upper row), isolectin GS-IB4 (green; middle row) and α-SMA (grey; lower row) included in Fig 2B. (PDF) [file pone.0173927.s003.pdf]

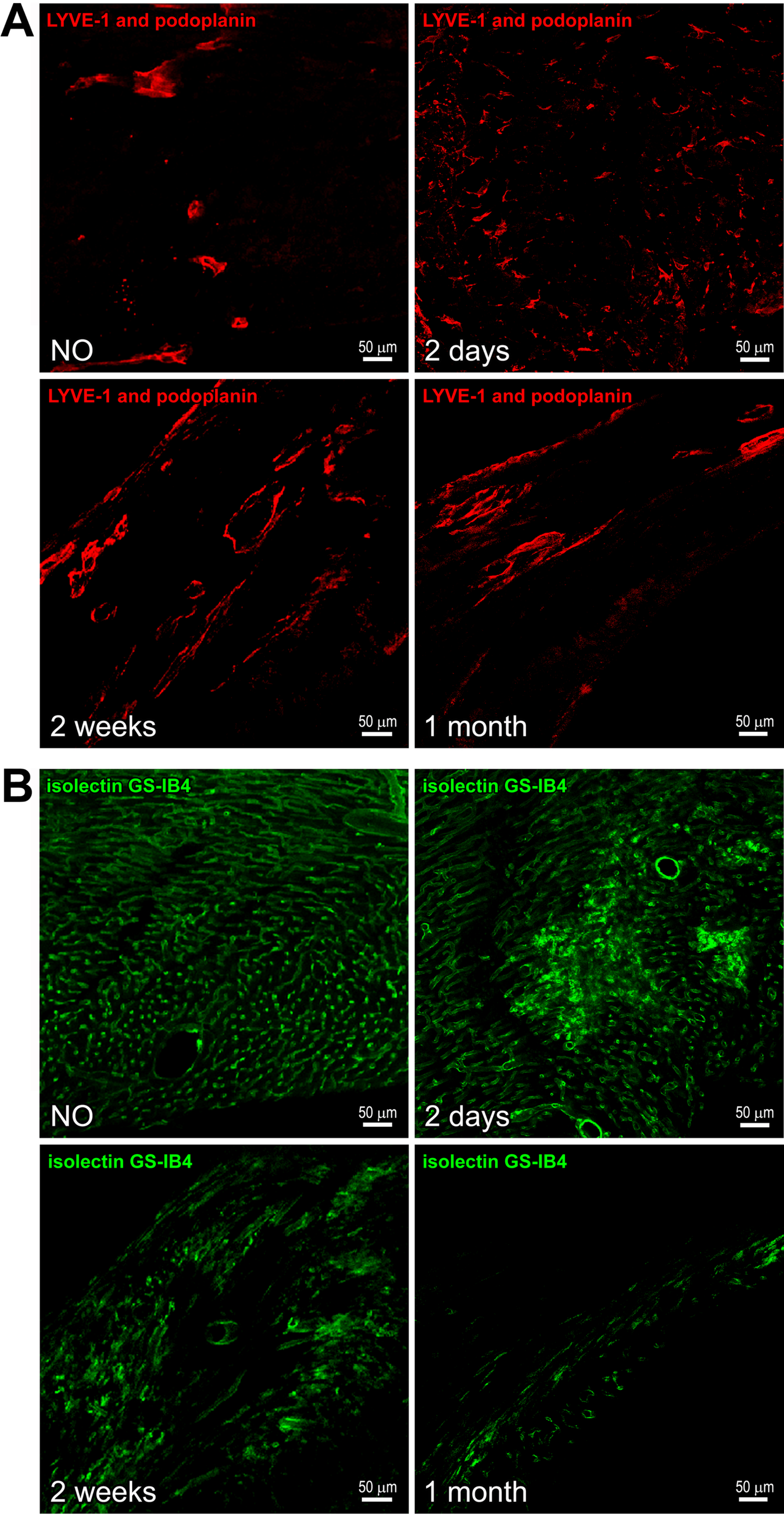
A
LYVE-1 and podoplanin
NO
50 μm
LYVE-1 and podoplanin
2 days
50 μm
LYVE-1 and podoplanin
2 weeks
50 μm
LYVE-1 and podoplanin
1 month
50 μm
B
isolectin GS-IB4
NO
50 μm
isolectin GS-IB4
2 days
50 μm
isolectin GS-IB4
2 weeks
50 μm
isolectin GS-IB4
1 month
50 μm
