# Supplementary material for: Phenotypically heterogeneous podoplanin-expressing cell populations are associated with the lymphatic vessel growth and fibrogenic responses in the acutely and chronically infarcted myocardium
Source: PLoS One. 2017 Mar 23;12(3):e0173927. doi: 10.1371/journal.pone.0173927 (PMC5363820; doi:10.1371/journal.pone.0173927)
Supplement: S3 Fig — (A,B) Isolated cardiac cells were co-stained with podoplanin and the indicated antibodies. (A) LYVE-1. Representative scatterplots are shown. Numbers indicate proportions of LYVE-1-positive cells within total podoplanin-positive populations (calculated as % cells in Q2 out of the sum of Q1 and Q2). Samples labeled with non-immune IgGs (IgGs) and podoplanin only or LYVE-1 only were used to determine the gates and calculate background. (B) Frequency (%) of podoplanin-positive cells co-labeled with PECAM-1, CD34, Prox-1, or VEGFR-3, respectively, was calculated as in (A). Graphs displaying values for the individual hearts and the respective means for each group (red lines) are shown. PECAM-1: ns, not significant; *P = 0.0001 by one-way ANOVA. CD34: ns, not significant for SHAM vs. MI by two-tailed t-test. Prox-1: *P = 0.0036 for SHAM vs. MI by two-tailed t-test. VEGFR-3: ns, not significant by one-way ANOVA. (PDF) [file pone.0173927.s004.pdf]

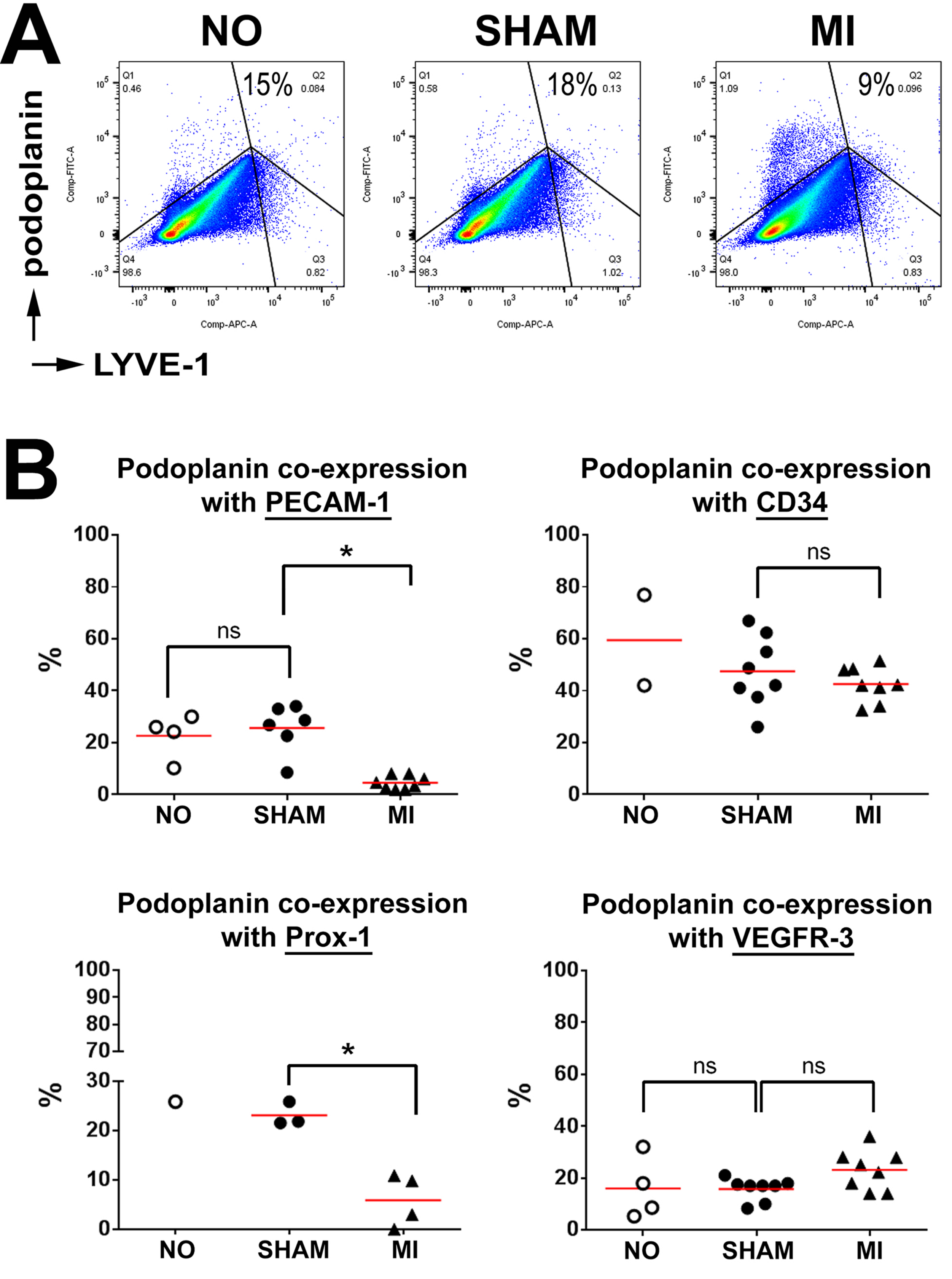
A
NO
SHAM
MI
podoplanin
LYVE-1
Comp-FITC-A
Comp-APC-A
Q1
0.46
15%
Q2
0.084
Q4
98.6
Q3
0.82
Q1
0.58
18%
Q2
0.13
Q4
98.3
Q3
1.02
Q1
1.09
9%
Q2
0.096
Q4
98.0
Q3
0.83
B
Podoplanin co-expression with PECAM-1
Podoplanin co-expression with CD34
Podoplanin co-expression with Prox-1
Podoplanin co-expression with VEGFR-3
%
ns
*
NO
SHAM
MI
0
20
40
60
80
100
