# Supplementary material for: Phenotypically heterogeneous podoplanin-expressing cell populations are associated with the lymphatic vessel growth and fibrogenic responses in the acutely and chronically infarcted myocardium
Source: PLoS One. 2017 Mar 23;12(3):e0173927. doi: 10.1371/journal.pone.0173927 (PMC5363820; doi:10.1371/journal.pone.0173927)
Supplement: S4 Fig — Representative scatterplots (left) and the summary graph (right) displaying individual values for each heart with the respective means of the frequency of podoplanin-positive cells co-labeled with PDGFRα or PDGFRβ are shown. Calculated as % cells in gate Q6 out of the sum of the gates Q5 and Q6. Samples labeled with non-immune IgGs (IgGs) and podoplanin only, or PDGFRα or PDGFRβ only (PDGFR only), were used to determine the gates and calculate background. (PDF) [file pone.0173927.s005.pdf]

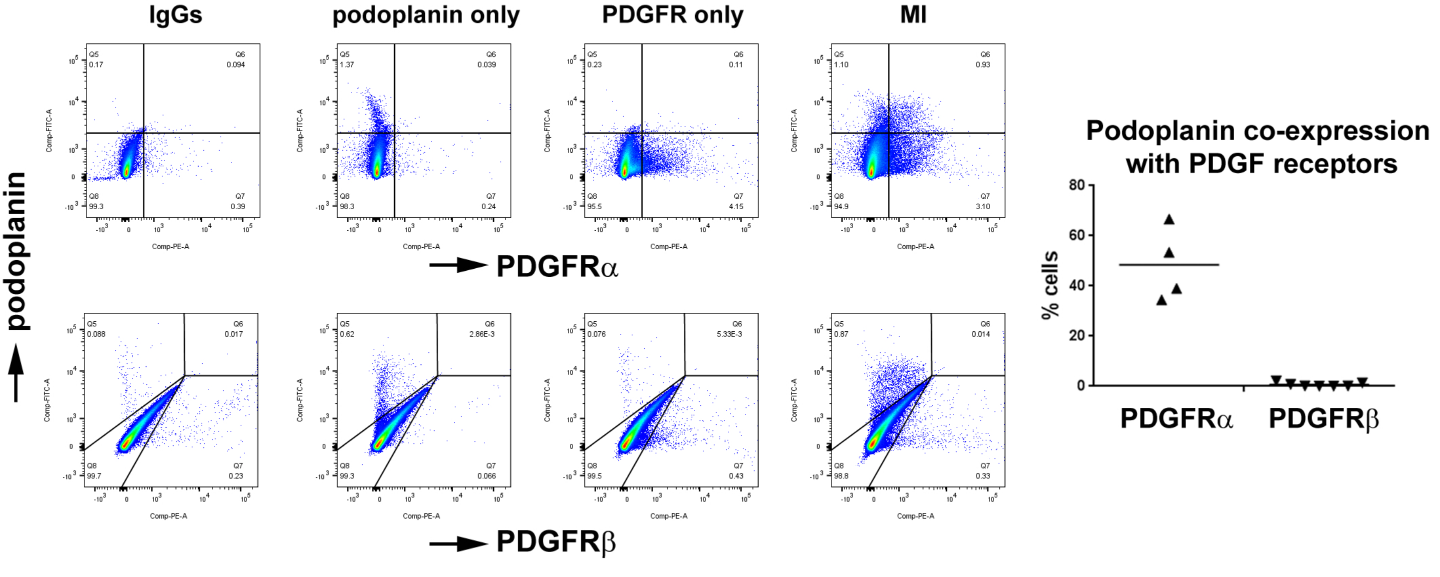

IgGs
podoplanin only
PDGFR only
MI
podoplanin
PDGFRα
PDGFRβ
Comp-FITC-A
Comp-PE-A
Q5 0.17
Q6 0.094
Q8 99.3
Q7 0.39
Q5 1.37
Q6 0.039
Q8 98.3
Q7 0.24
Q5 0.23
Q6 0.11
Q8 95.5
Q7 4.15
Q5 1.10
Q6 0.93
Q8 94.9
Q7 3.10
Q5 0.088
Q6 0.017
Q8 99.7
Q7 0.23
Q5 0.62
Q6 2.86E-3
Q8 99.3
Q7 0.066
Q5 0.076
Q6 5.33E-3
Q8 99.5
Q7 0.43
Q5 0.87
Q6 0.014
Q8 98.8
Q7 0.33
Podoplanin co-expression with PDGF receptors
% cells
80
60
40
20
0
PDGFRα
PDGFRβ
